# Comprehensive analysis of cuproptosis and copper homeostasis genotyping and related immune land scape in lung adenocarcinoma

**DOI:** 10.1038/s41598-023-43795-3

**Published:** 2023-10-02

**Authors:** Dayuan Luo, Xiang Wang, Wei Feng

**Affiliations:** 1 Department of Thoracic Surgery, Second Xiangya Hospital, Central South University, Changsha, 410013 Hunan China; 2 Department of Cardiothoracic Surgery, Third Xiangya Hospital, Central South University, Changsha, 410013 Hunan China

**Keywords:** Cancer, Computational biology and bioinformatics, Genetics

## Abstract

Cuproptosis is a manner of cell death which is related to the homeostasis of copper ions in the cellular environment and is expected to open a new direction of anti-tumor therapy. However, the studies on cuproptosis and copper homeostasis in lung adenocarcinoma (LUAD) are still limited. In this study, we identified new cuproptosis and copper homeostasis related genes (CHRGs) which were effective in stratifying genotyping clusters with survival differences based on transcriptomic data obtained from The Cancer Genome Atlas (TCGA) and Gene Expression Omnibus (GEO). Weighted Gene Co-expression Network Analysis (WGCNA) further expands the screening boundary of CHRGs, and finally we established a 10-CHRGs-based prognostic signature using lasso-penalized cox regression method, which were validated in GSE30219. Comprehensive bioinformatics analysis revealed these genes are potential regulators of modulating immunotherapy efficacy, drug resistance, tumor microenvironment infiltration, and tumor mutation patterns. Lastly, the scRNA-seq datasets GSE183219 and GSE203360 offers the evidences that CHRGs signature are mainly distributed in cancer epithelial cells, real time quantitative polymerase chain reaction (RT-qPCR) also confirmed the differential expression of these genes between normal lung cell line and lung adenocarcinoma cell lines. Collectively, our findings revealed new cuproptosis and copper homeostasis related genotyping clusters and genes which may play important roles in predicting prognosis, influencing tumor microenvironment and drug efficacy in LUAD patients.

## Introduction

According to global incidence and deaths in recent report for total cancers and 29 cancer groups, the cancer of tracheal, bronchus, and lung had the highest morbidity and mortality in the world, and lung adenocarcinoma (LUAD) accounts for the largest number of patients in lung cancer^1^. However, only a small number of LUAD patients can be found in the early stage and receive surgical or medical treatment, beyond that, drug resistance is also a serious problem in drug-oriented comprehensive treatment^2,3^. In light of these challenging issues, it is crucial to explore novel therapeutic targets and identify disease markers that are associated with prognosis and drug sensitivity.

Cuproptosis is another manner of death associated with metal ions, discovered subsequent to ferroptosis^4–6^. Copper ion is an important cofactor of enzymes in cells and humans, but homeostasis and concentration overload of copper can lead to oxidative stress and cytotoxicity. Available evidence suggests that excess copper can bind to the lipids components of the tricarboxylic acid (TCA). The subsequent accumulation of these copper-bound lipoylated mitochondrial proteins and the loss of the Fe-S cluster protein trigger proteotoxic stress, which manifests as a distinct manner of cell death^7,8^. Cuproptosis is closely related to copper homeostasis in cells, from prokaryotes to eukaryotes, the maintenance of copper homeostasis is fine-regulated mainly by preventing excessive accumulation of copper in cells from causing cell death. Tsvetkov et al.^4^ also suggested that the mechanism of copper ionophore-induced cell death involved the accumulation of copper in cells, rather than the action of small molecule chaperones themselves. Therefore, studying only the cuproptosis process without considering the impact of copper ion homeostasis-related genes on cancer may be insufficient.

Bioinformatics analysis technology enables the exploration of potential disease-related genes at a broader and more comprehensive scale, leveraging big data^9^. Due to the limited number of bioinformatics studies on the combined analysis of cuproptosis and copper homeostasis, we fully explored disease-related genes potentially related to cuproptosis and copper homeostasis function through K-means clustering algorithm of unsupervised machine learning methods and Weighted Gene Co-expression Network Analysis (WGCNA), finally developed a new 10-gene risk signature using comprehensive bioinformatics methods based on cuproptosis and copper homeostasis related genes (CHRGs), which can be helpful for the prediction of prognosis, immunotherapy efficacy and the selection of antitumor drugs for LUAD patients. The biological significance of CHRGs in LUAD and its effects on tumor microenvironment, immune infiltration level and tumor mutation patterns were systematically explored.

## Results

### Identification of cuproptosis subtypes and copper homeostasis subgroups

**Figure 1:**
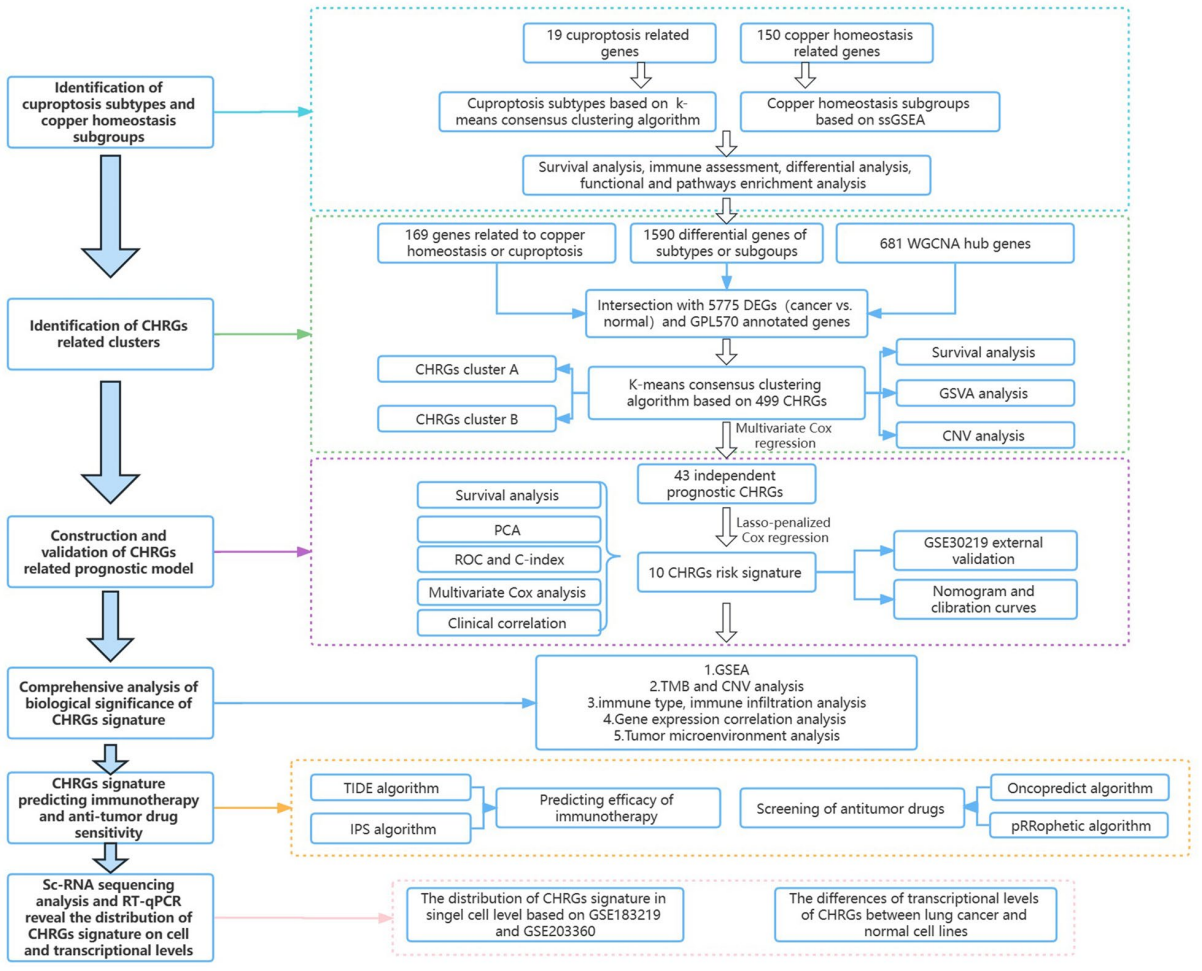
The specific workflow graph of data analysis.

**Figure 2:**
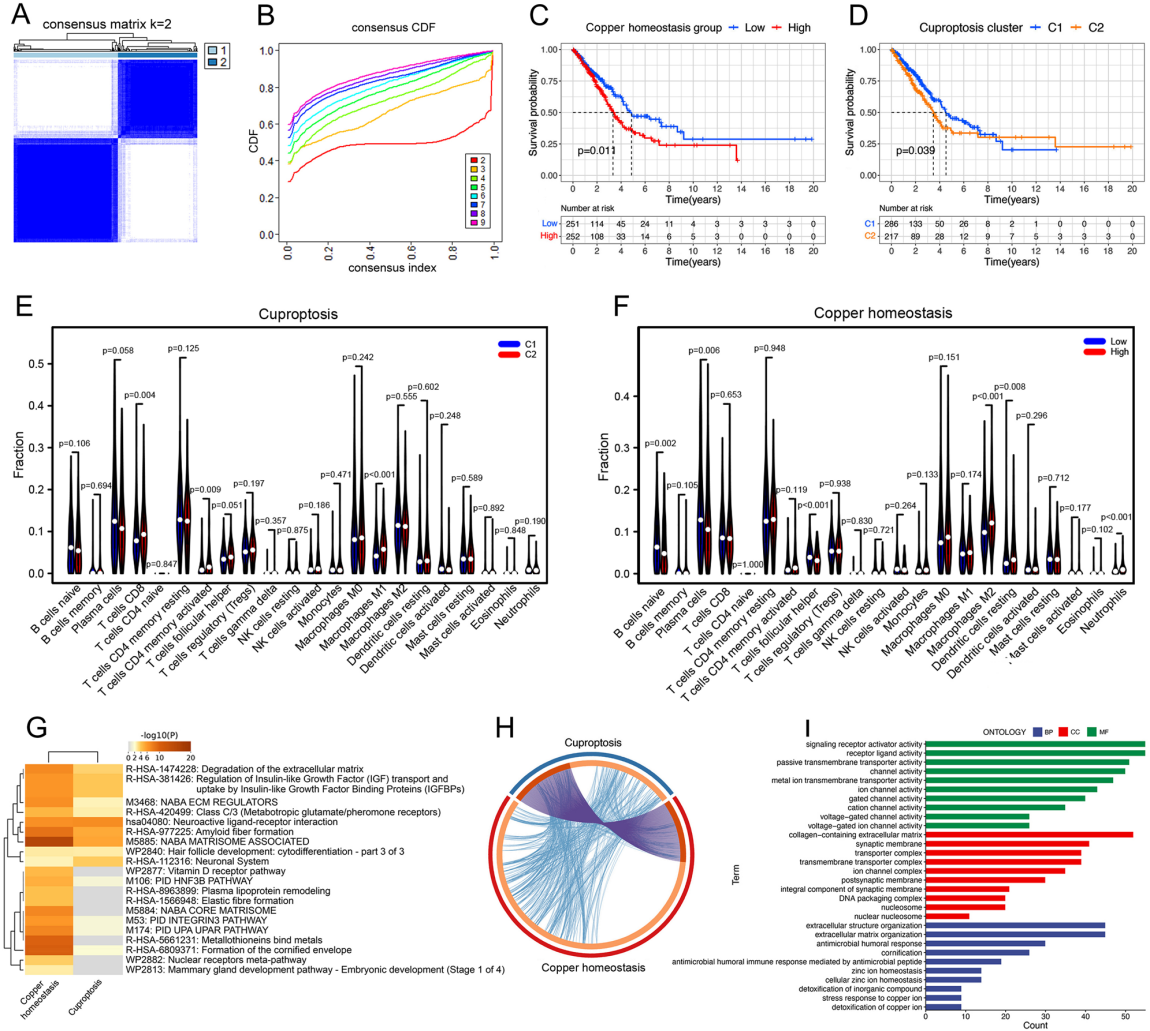
Identification of cuproptosis subtypes and copper homeostasis subgroups. (**A, B**) Based on the K-means clustering algorithm, 503 lung cancer samples can be divided into two subtypes based on 19 cuproptosis related genes when the k value is 2. (**C, D**) Survival differences between cuproptosis subtypes (**C**) and between copper homeostasis sub groups(**D**). (**E, F**) The differences of immune cell relative content between cuproptosis subtypes (**E**) and between copper homeostasis subgroups (**F**) based on CIBERSORT. (**G**) The pathway enrichment analysis of the differential genes between cuproptosis subtypes and between copper homeostasis subgroups based on Metascape database. (**H**) The genetic overlap between the two kinds of differential genes. (**I**) Gene Ontology(GO) function enrichment analysis of differential genes between cuproptosis subtypes and between copper homeostasis subgroups.

The research process for this study can be outlined by the flow chart shown in Fig. 1. When k values equal to 2, the 503 samples could be clearly divided into two subtypes named C1 and C2 based on 19 cuproptosis genes using k-means consensus clustering algorithm (Fig. 2A, B). We calculated the copper homeostasis score of each sample using the ssGSEA algorithm based on 150 genes from 14 copper homeostasis pathways. Samples were then categorized into high and low groups based on the median score. Our analysis revealed significant survival differences between cuproptosis subtypes and copper homeostasis subgroups (Fig. 2C, D). There is significant differences between the two cuproptosis subtypes in CD8+ T cells and M1 macrophages (Fig. 2E), while the copper homeostasis subgroups mainly had significant differences in naive B cell, neutrophils, follicular helper T cell and M2 macrophages (Fig. 2F).

In order to explore the biological significance further, we identified 798 differential genes between cuproptosis subtypes and 2304 differential genes between copper homeostasis subgroups, there is a considerable number of genetic intersections (Fig. 2H). After the removal of duplicates, total 1590 differential genes were finally obtained for subsequent analysis. These genes were co-enriched in the degradation of extracellular matrix and the regulation of insulin-like growth factor protein binding pathways, and the functions of activity and transport capacity of metal ion transmembrane, including copper ions. In addition, they were significantly enriched in DNA packaging, humoral immune response and detoxification function of inorganic compounds such as copper (Fig. 2G, I). These results suggest the differential genes derived from cuproptosis subtypes and copper homeostasis subgroups may have important effects on the copper-related intracellular environment.

### Identification of CHRGs related clusters

**Figure 3:**
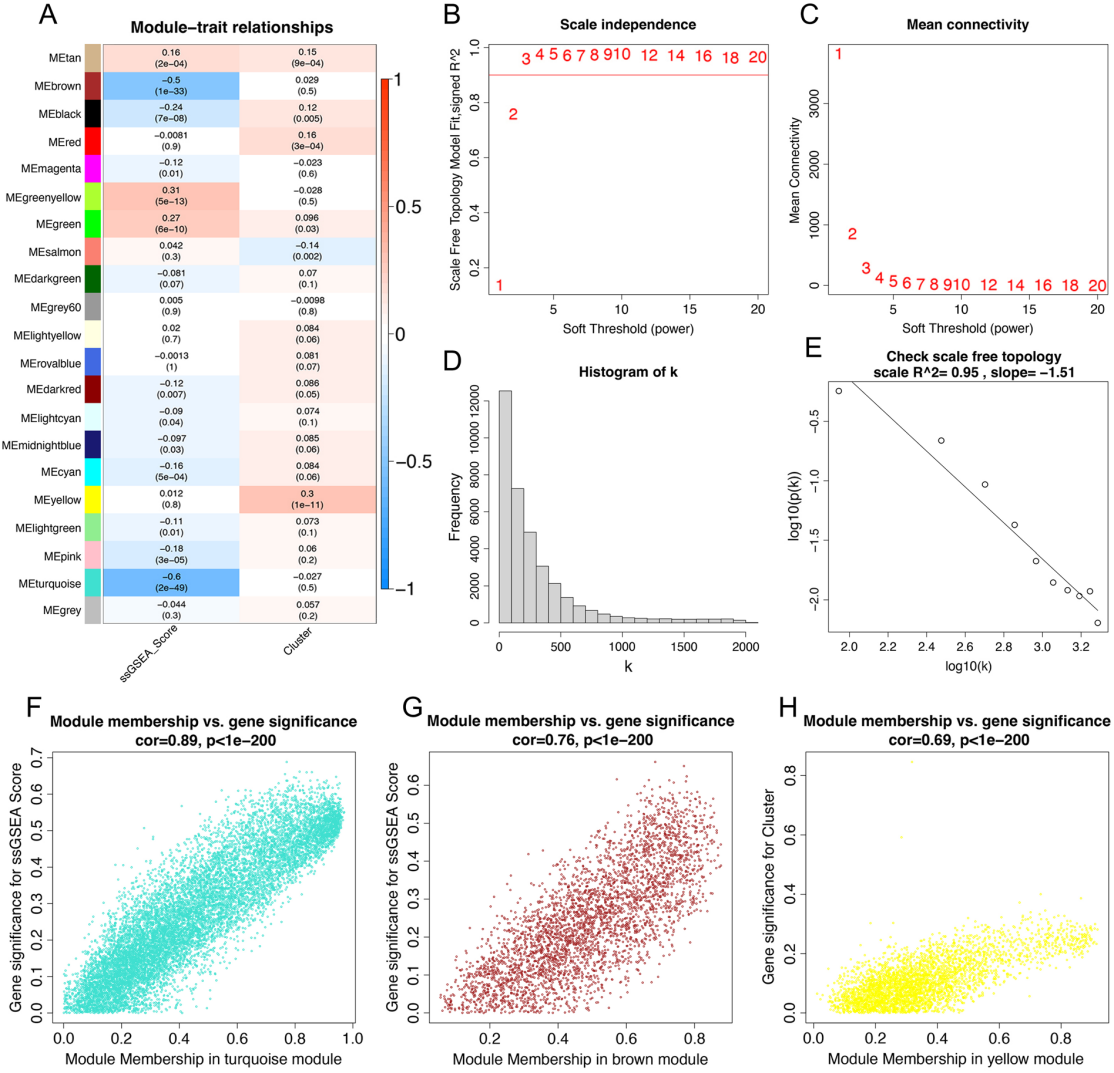
Weighted gene co-expression network and hub gene identification. (**A**) The correlation between gene consensus modules and copper homeostasis scores and cuproptosis subtypes. (**B**) The correlation between soft treshold and R^2^. (**C**) Mean connectivity under different soft treshold values. (**D, E**) log(k), the logarithm of the number of nodes whose node degree is k (**D**), is negatively correlated with the probability logarithm '(log(p(k)))' of the occurrence of this node, which conforms to the Scale-free network (**E**). (**F**) Correlation coefficients of Turquoise module (**F**) and Brown module (**G**) with copper homeostasis. (**H**) Correlation coefficient between Yellow module and cuproptosis subgroups.

**Figure 4:**
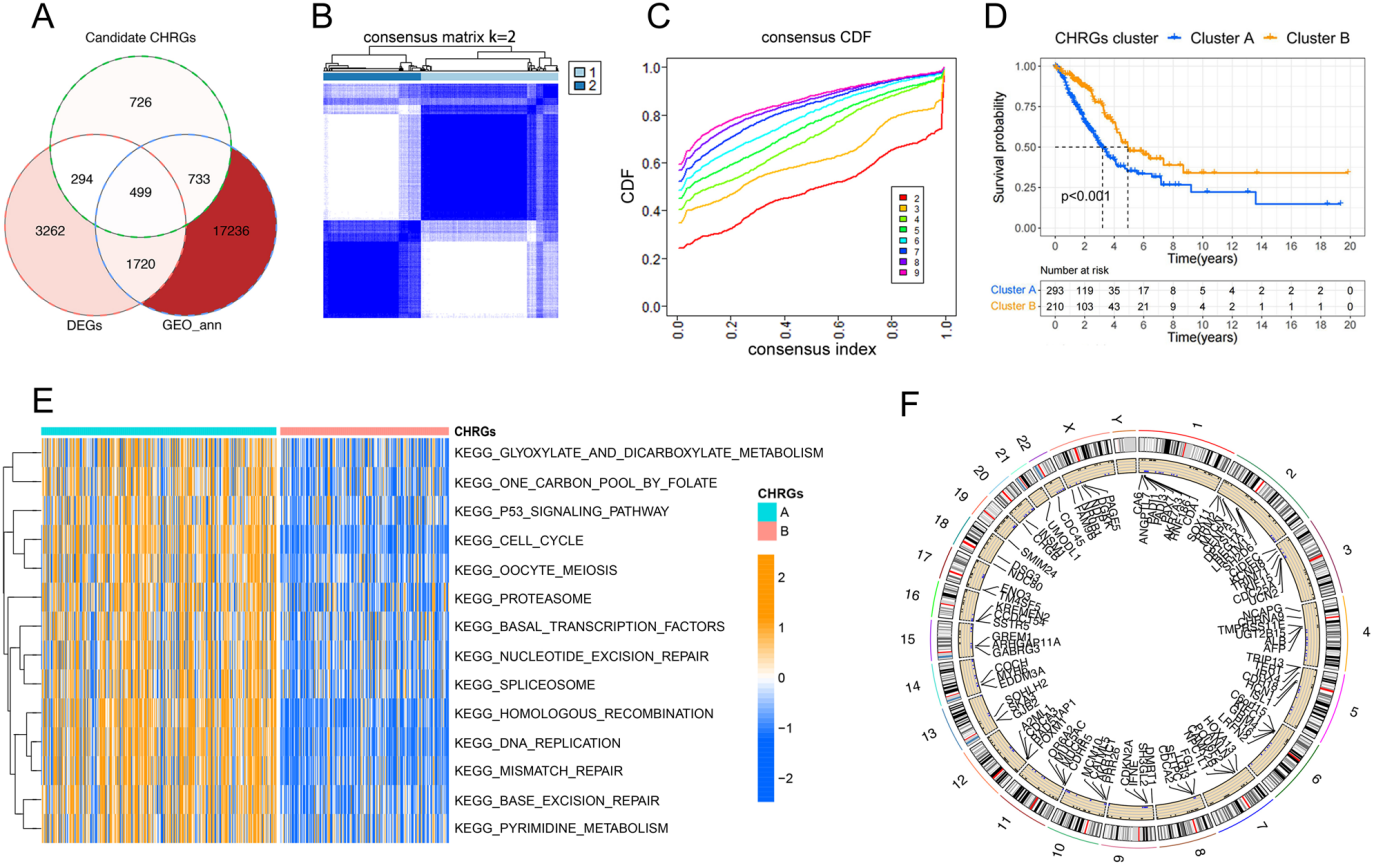
Identifiction of CHRGs related clusters. (**A**) There were 499 gene intersections among candidate CHRGs, DEGs, and annotated genes from GPL570. (**B, C**) Based on k-means clustering algorithm, 503 lung cancer samples could be divided into cluster A an cluster B based on 499 CHRGs. (**D**) KM analysis showed significant survival differences between CHRGs clusters. (**E**) Gene Set Variation Analysis (GSVA) enrichment analysis of CHRGs clusters. (**F**) Copy number variation of 499 CHRGs on each chromosome.

To identify additional genes associated with cuproptosis and copper homeostasis, we successfully constructed a scale-free network matrix using WGCNA with all mRNA and LncRNA data from 503 lung adenocarcinoma samples (Fig. 3B–E). The correlation matrix revealed that the Turquoise, Brown, and Yellow modules showed a strong correlation with cuproptosis and copper homeostasis. These modules include a total of 681 hub genes (Fig. 3A, F–H). After merging 1590 differential genes, 681 hub genes, 169 cuproptosis or copper homeostasis related genes, while removing duplicates, we obtained 2252 genes as candidate CHRGs. Following the performance of differential analysis on LUAD samples and normal samples from the TCGA cohort, a total of 5775 differentially expressed genes (DEGs) were obtained. Subsequently, by intersecting the DEGs with the candidate CHRGs and annotated genes based on GPL570, we identified 499 genes for further analysis (Fig. 4A).

Based on the 499 genes identified as hub genes related to copper homeostasis and cuproptosis function, we employed the k-means consensus clustering algorithm to successfully categorize 503 samples into two distinct clusters, namely cluster A and cluster B (Fig. 4B, C). Notably, Kaplan–Meier analysis revealed significant survival differences between these two clusters (Fig. 4D). GSVA indicated that Cluster A was significantly enriched in cell cycle, DNA replication, nucleotide resection and repair, p53 pathway, etc., while Cluster B was extremely low in activity in these pathways (Fig. 4E). The observation from Fig. 4F reveals that the 499 CHRGs exhibit pronounced copy number variations, particularly concentrated on chromosomes X, 5, 11, and 12. This indicates a genomic instability of the CHRGs. These findings imply that Cluster A, which is closely associated with tumor occurrence and development, is potentially impacted by the 499 CHRGs. Furthermore, this suggests that these genes may have a significant influence on the prognosis of LUAD.

### Construction and validation of CHRGs related prognostic model

**Figure 5:**
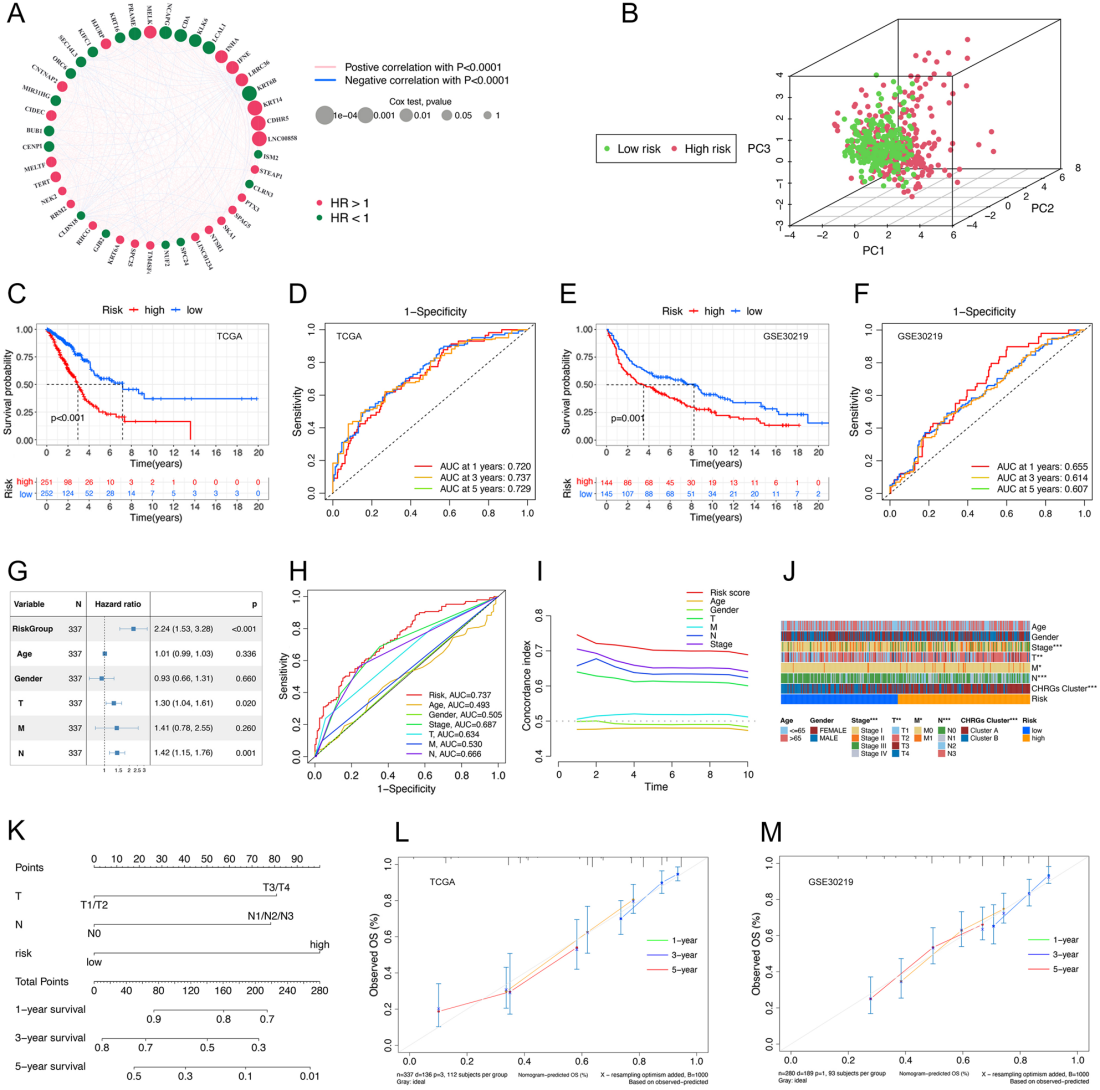
Construction and validation of CHRGs related prognostic model. (**A**) 43 independent prognostic CHRGs for OS of LUAD. (**B**) PCA for 503 samples based on CHRGs signature. (**C, E**) KM analysis shows significant survival difference between risk groups in TCGA cohort (**C**) and GSE30219 (**E**). (**D, F**) The AUC of CHRGs signature for 1, 3 and 5 years in TCGA cohort (**D**) and GSE30219 (**F**). (**G**) Forest map shows risk group and some clinical factors are independent prognostic signature in TCGA cohort based on multivariate regression analysis. (**H**) The AUC of CHRGs signature and clinical factors for 1, 3 and 5 years survival in TCGA cohort. (**I**) C-index of risk group and clinical factors in TCGA cohort (1000 randomizations). (**J**) Heatmap of correlation among risk group and clinical factors. (**K**) Nomogram with indicators of risk group, T and N. (**L, M**) Calibration curves for nomogram in TCGA cohort (**L**) and GSE30219 (**M**).

To identify key prognostic CHRGs, we performed multivariate Cox regression analysis on 499 CHRGs, among which 43 genes were independent prognostic factors for overall survival (OS) (Fig. 5A). 15 genes corresponding to ‘lambda.1se’ were selected as candidate genes for the risk model by LASSO regression (Supple Fig. 1). According to stepwise multi-Cox regression and AIC, 10 genes were eventually included in Cox proportional hazard model, which could best interpret and fit data with the least parameters. 10 genes as follows: *LINC00858*, *INHA*, *LCAL1*, *SEC14L3*, *CNTNAP2*, *MELTF*, *RHCG*, *TM4SF4*, *NTSR1*, *PTX3* (Supple Table 1). Subsequently, we calculated risk scores for each sample according to the following formula:

$$risk\;{ }score = { }\sum \left( {\beta_{{i{ }}} \times Exp_{i} } \right)$$

The samples were categorized into high-risk and low-risk groups based on the median risk score, and distinguished using Principal Component Analysis (PCA) (Fig. 5B). Significant survival differences existed between two risk groups in the TCGA cohort (median risk score: 0.957) and external data set GSE30219 (median risk score: 5.96) (Fig. 5C, E). Multivariate Cox analysis showed that T stage (p-value = 0.02, HR = 1.30, CI: [1.04, 1.61]), N stage (p-value = 0.001, HR = 1.42, CI: [1.15, 1.76]) and risk group (p-value < 0.001, HR = 2.24, CI: [1.53, 3.28]) were independent prognostic factors for OS (Fig. 5G). The AUC of CHRGs signature for 1, 3 and 5 years were 0.720, 0.737 and 0.729 respectively (Fig. 5D), significantly higher than that of tumor clinical factors (Stage: AUC = 0.687, T: AUC = 0.634, N: AUC = 0.666) (Fig. 5H). Furthermore, the risk signature demonstrated the highest C-index among 1000 random repetitions(Fig. 5I). The CHRGs signature’s predictive ability was well confirmed in the external microarray chip dataset GSE30219, with AUC values of 0.655, 0.614, and 0.607 for 1, 3, and 5-year survival, respectively. This indicates that the CHRGs signature has robust universality (Fig. 5F). In the TCGA cohort, the risk groups were significantly associated with tumor stage, T stage, and N stage (Fig. 5J). A nomogram was constructed based on the independent prognostic factors (T stage, N stage) and the CHRGs risk signature using 337 LUAD samples from TCGA (Fig. 5K). The nomogram’s performance was validated using calibration curves in both the TCGA cohort and GSE30219 dataset(Fig. 5L, M).

### Comprehensive analysis of biological significance of CHRGs signature

**Figure 6:**
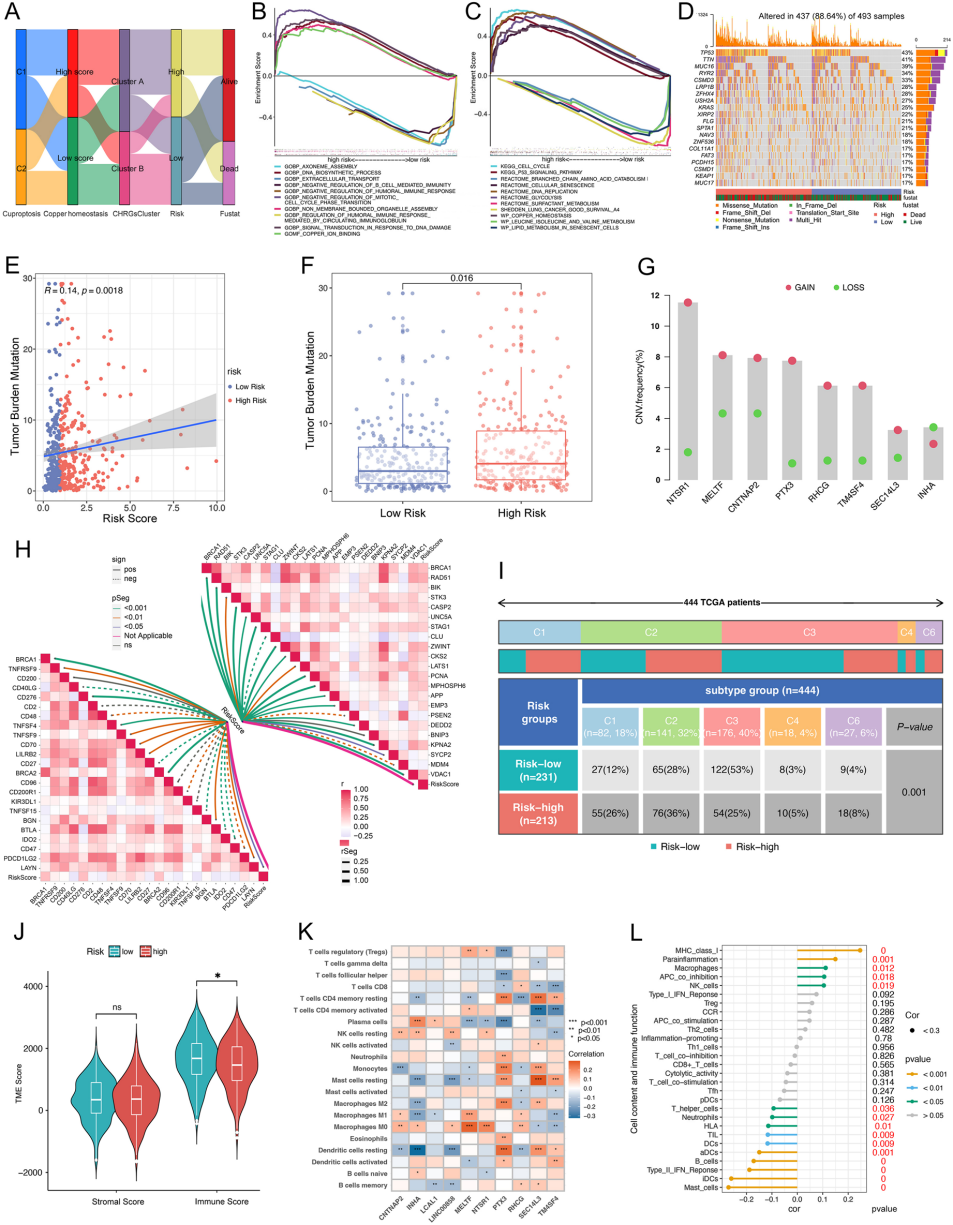
Comprehensive analysis of biological significance of CHRGs signature. (**A**) Sample distribution relationship among cuproptosis subtypes, copper homeostasis subgroups, CHRGs clusters, risk groups and prognosis. (**B, C**) GSEA functional enrichment analysis (**B**) and pathway enrichment analysis (**C**) for different risk groups. (**D**) Tumor mutation rate, major mutant genes in TCGA cohort. (**E**) Risk score was positively correlated with TMB based on Spearman analysis. (**F**) The diffrence of mean TMB between high- and low- risk group. (**G**) Copy number variation of 8 CHRGs in LUAD samples. (**H**) The correlations among risk score and the expression levels of genes related to immune checkpoint, cell cycle and apoptosis based on Spearman analysis. (**I**) Distribution of immune types in defferent risk groups. (**J**) Tumor microenvironment differences between risk groups. (**K-L**) Correlation between CHRGs and immune cell relative content calculated by CIBERSORT based on Spearman analysis. (**L**) Correlations of risk score with the infiltration levels of immune cells and activitys of immune functions calculated by ssGSEA.

The high score subgroup of copper homeostasis is mainly concentrated in CHRGs Cluster A, which is mainly concentrated in the high-risk group and is associated with the worse prognosis of LUAD (Fig. 6A). The GSEA results showed that the high-risk group was significantly enriched in biological functions such as copper ion binding, DNA damage and synthesis reaction, and the pathways of copper homeostasis, cell cycle, p53 signaling, cell senescence, glycolysis, etc. While the low-risk group was mainly enriched in immune response, B-cell response, amino acid and lipid metabolism (Fig. 6B, C). Interestingly, among 19 cuproptosis related genes, there were 6 of which expression were significantly related to the risk groups and all significantly up-regulated in the high-risk group (Supple Fig. 2). The findings indicate a potentially stronger association between the high-risk group and the processes of cuproptosis and copper homeostasis.

We further investigated the differences in immune response-related indicators between the identified risk groups. According to the somatic mutation data of 493 LUAD samples, 88.64% patients had gene mutation events and the mutation rate was higher in the high-risk group (Fig. 6D). There was a positive correlation between tumor mutation burden (TMB) and risk score (Fig. 6E, F). The 8 intersections within the CHRGs signature exhibited varying numbers of copy number variation (CNV) events, which suggests genetic instability within these regions (Fig. 6G). Additionally, We found multiple immune checkpoints related genes, including BTLA and CD27, were significantly correlated with risk scores, as well as multiple cell cycle or apoptosis-related genes which mostly showed positive correlation (Fig. 6H). In the tumor microenvironment, the immune cell score of the low-risk group is significantly higher (Fig. 6J). CIBERSORT analysis revealed that a majority of CHRGs exhibited correlations with the abundance of various immune cells, such as M0 macrophages and CD4+ T memory resting cells (Fig. 6K). SsGSEA further shows risk score was positively correlated with the content of NK cell, macrophage, the function of MHC I and APC co inhibition, while negatively correlated with B cell content and function of HLA (Fig. 6L). The distribution of immune types differed significantly between the low-risk and high-risk groups (Fig. 6I). The low-risk group was mainly associated with the C3 inflammatory response type, which indicates immune balance and a good prognosis for LUAD. In contrast, the high-risk group was predominantly associated with the C1 wound Healing and C2 IFN-γ dependent types, which are associated with angiogenesis, tumor invasion, and a worse prognosis in lung cancer.

### CHRGs signature predicts immunotherapy efficacy and anti-tumor drug sensitivity

**Figure 7:**
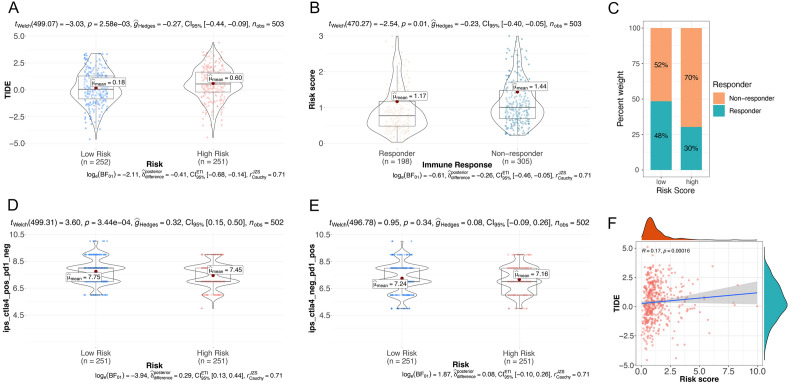
CHRGs signature predicts immunotherapy efficacy. (**A**) Difference of mean TIDE score between different risk groups. (**B**) Difference of mean risk score between Responder group and Non-Responder group. (**C**) Proportion of Responder and Non-Responder predicted by the TIDE database in different risk groups. (**D**) Difference of mean IPS score between different risk groups with positive response to CTLA-4 inhibitor treatment. (**E**) Difference of mean IPS score between different risk groups with positive response to PD-1 inhibitor treatment. (**F**) Risk score was positively correlated with TIDE score based on Spearman analysis.

**Figure 8:**
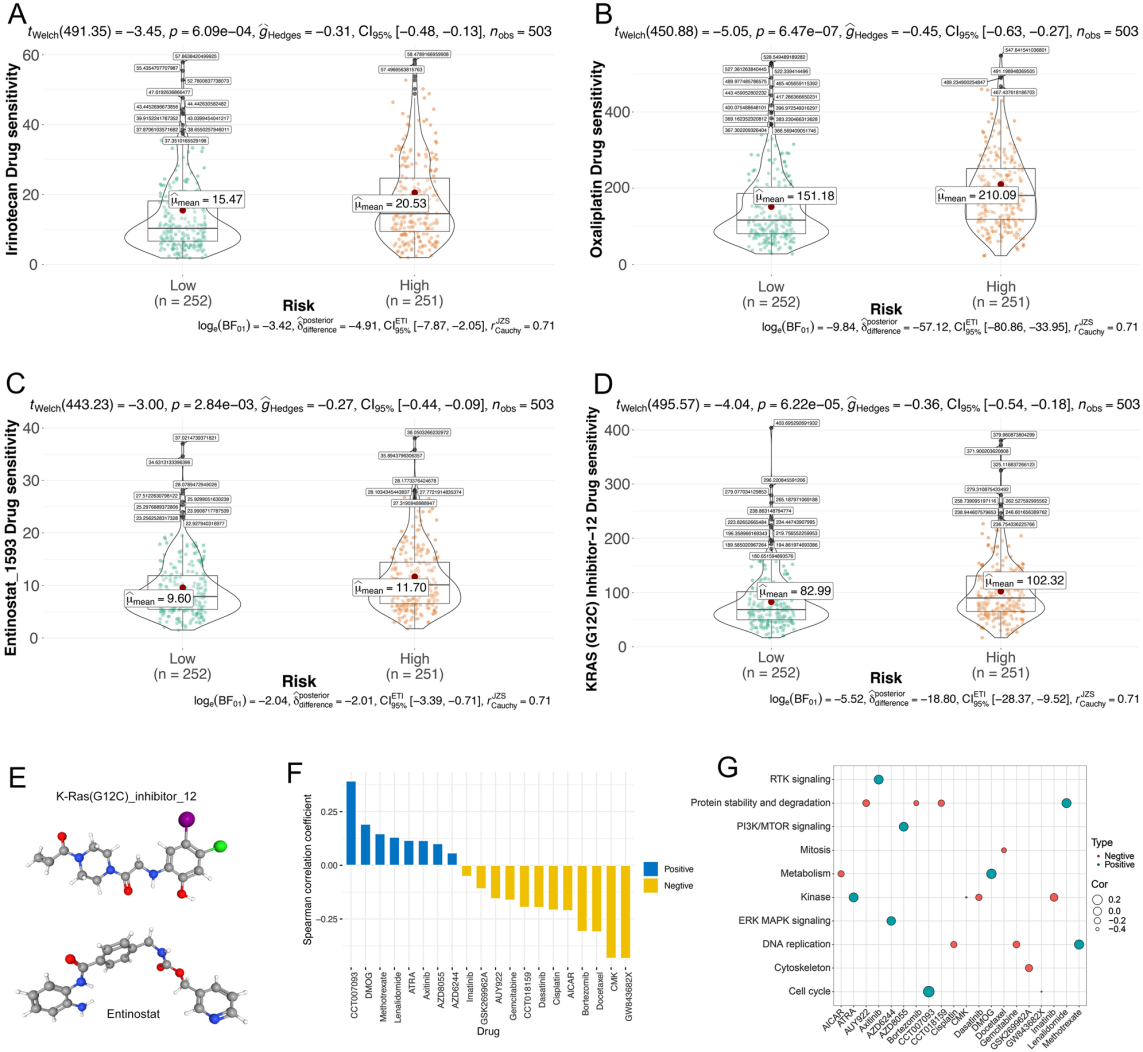
CHRGs signature predicts anti-tumor drug sensitivity. (**A–D**) Drug sensitivity differences of irinotecan (**A**), oxaliplatin (**B**), Entinostat(**C**), and KRAS intel-12 (**D**) in different risk groups (based on R-package ‘Oncopredict’). (**E**) Molecular structures of Entinostat and KRAS intel-12 obtained from PubChem database. (**F**) Spearman correlation coefficients of risk score and IC 50 values of 20 antitumor drugs (based on R-package ‘pRRophetic’). (**G**) Target pathways of 20 antitumor drugs (based on R package 'pRRophetic').

We hypothesize that the observed significant variances in tumor mutation burden (TMB), expression levels of immune checkpoint related genes, immune types, and immunoinfiltration levels among the risk groups may denote their potential for predicting the efficacy of immunotherapy. First, we analyzed the immune score of the TCGA lung adenocarcinoma cohort and the predictive response for immune checkpoint in TIDE database. The findings revealed a significant elevation in TIDE score within the high-risk group (Fig. 7A), and a positive correlation between risk score and TIDE score. This correlation suggests that the high-risk group is more prone to immune escape during immune checkpoint inhibitor treatment, resulting in a higher proportion of non-responders within the high-risk group (Fig. 7B, C, F). Another immune scoring algorithm, IPS, suggests that patients in the low-risk group may tend to a better response when using CTLA-4 immune checkpoint inhibitors (Fig. 7D, E). We employed the R-package ‘Oncopredict’ to compute the sensitivity of various antitumor drugs for each sample. Through this analysis, we identified two chemotherapy drugs, irinotecan and oxaliplatin, along with two promising candidate drugs, Entinostat and KRAS inoperation-12, for NSCLC. These drugs were selected based on their higher sensitivity in patients belonging to the low-risk group (Fig. 8A–E). Their selection was informed by data sourced from the PubChem database (https://pubchem.ncbi.nlm.nih.gov/, accessed on 17 November 2022). In addition, we calculated the 50% inhibiting concentration (IC50) of drugs using R-package ‘pRRophetic’ and chosen 20 antitumor drugs with significant correlation with risk score (p-value < 0.05) based on Spearman analysis. Consequently, the risk score exhibited negative sensitivity towards 8 drugs, including DMOG and Axitinib, while demonstrating a positive correlation with sensitivity towards 12 drugs, such as cisplatin, docetaxel, CMK, and others (Fig. 8F). Among them, GW843682X, CMK, CCT007093 exhibited the strongest correlation. Furthermore, we explored the signaling pathways targeted by the selected drugs. We uncovered that the relationship between drug IC50 and risk scores based on RTK signaling, cell cycle and PI3K/mTOR signaling was positive. In contrast, drugs with a IC50 that was negatively related to the risk score targeted the DNA replication, protein stability and degradation (Fig. 8G). In conclusion, the establishment of the risk score will facilitate the exploration of the correct and effective treatment strategy.

### Sc-RNA sequencing analysis and RT-qPCR reveal the distribution of CHRGs signature on cell and transcription levels

**Figure 9:**
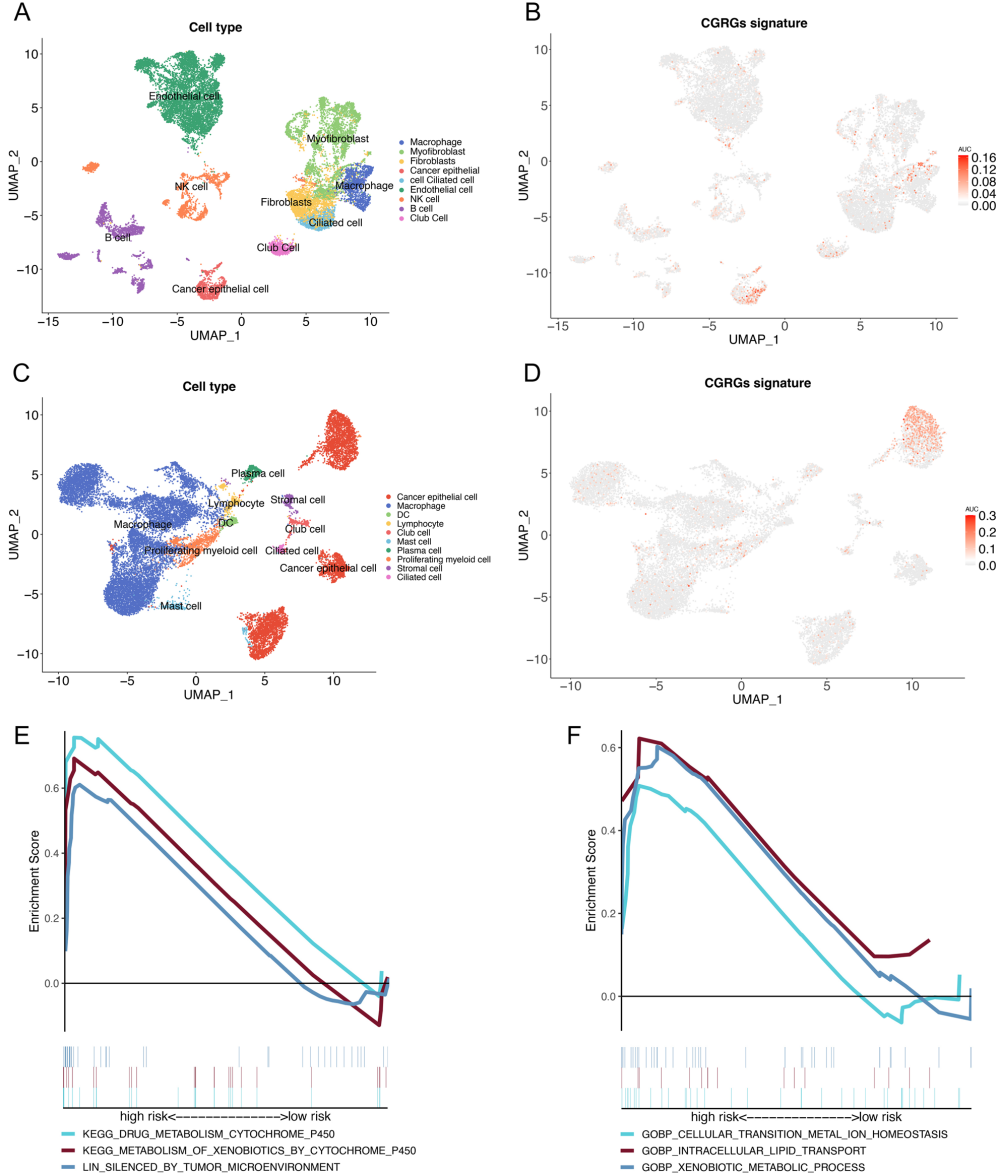
Sc-RNA sequencing analysis reveal the distribution of CHRGs signature on single cell level. (**A**, **C**) The annotated cell types of GSE183129 (**A**) and GSE203360 (**C**) based on UMAP. (**B, D**) The distribution of CHRGs signature of GSE183129 (**B**) and GSE203360 (**D**) were revealed based on UMAP. (**E, F**) GSEA revealed the enrichment pathways (**E**) and functions (**F**) of cells with high CHRGs distribution level.

**Figure 10:**
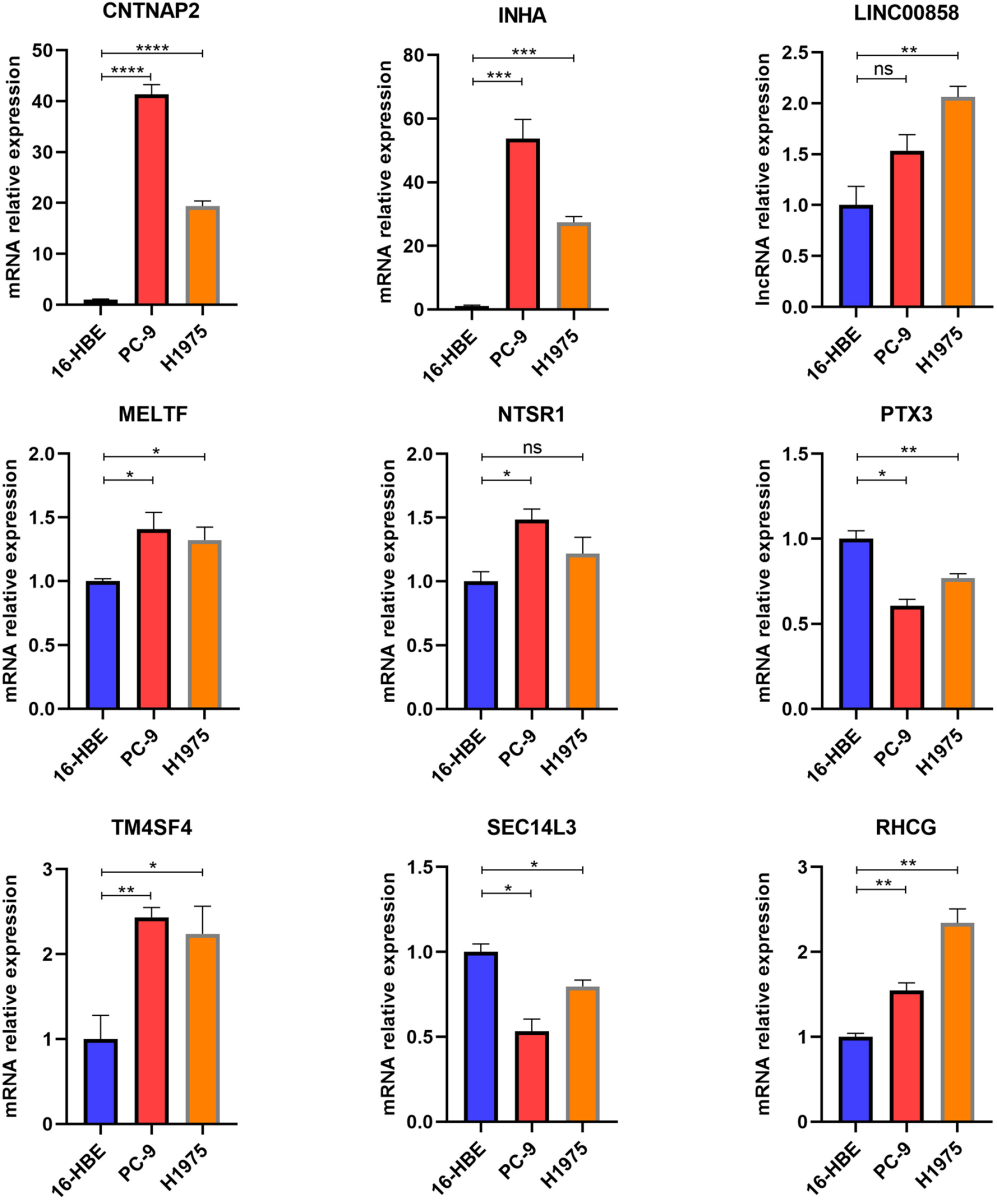
RT-qPCR reveal the differences of transcriptional levels of CHRGs signature between normal lung cell line (16-HBE) and adenocarcinoma cell line (PC-9 and H1975). Statistics were considered significant when the *p-value was less than 0.05, **p-value less than 0.01, ***p-value less than 0.001 and ****p-value less than 0.0001.

To assess the distribution of CHRGs signature on cell level, we analyzed 43,869 cells derived from 15 tumor samples in GSE183219 and 19,391 cells derived from 3 pairs of lung cancer and para-cancer samples in GSE203360. After the unqualified cells were filtered out, 19,471 cells and 19,030 cells respectively were retained for subsequent analysis. Upon completion of cell classification and annotation, we conducted a subsequent analysis to examine the distribution of CHRGs across various cell types (Fig. 9A, C). According to the “AUCcell” algorithm, the AUC thresholds of the two datasets were 0.017 and 0.030 respectively, with 1274 and 1337 cells having AUC above the threshold. These CHRGs enriched cells are mainly distributed in cancer epithelial cells, followed by macrophages (Fig. 9B, D). GSEA was performed on the cluster of cancer epithelial cell, we found that CHRGs enriched cells were mainly enriched in annotated pathways of P450-related drug metabolism and silenced tumor microenvironment, as well as the functions of cellular transition metal ions homeostasis, intracellular lipid transport and xenobiotic metabolic process (Fig. 9E, F). Based on RT-qPCR analysis, we verified the differences of transcription levels of 9 CHRGs between normal lung cell line and LUAD cell lines, excluding one lncRNA (LCAL1), which was not suitable for primer amplification of short fragments (Fig. 10).

## Discussion

To date, several studies have investigated the prognostic significance of previously identified cuproptosis-related genes in various types of cancers. However, there is still a need to identify and explore numerous new and potential genes that may be associated with cuproptosis^10,11^. In our study, we identified novel genotyping clusters related to cuproptosis and copper homeostasis using the classical machine learning method, K-means clustering algorithm, and further screened out modules with similar functions by weighted gene co-expression network analysis (WGCNA), from which we obtained CHRGs related to the prognosis of lung adenocarcinoma, and finally verified their differential expression level in cell lines and distributions in single cell level. The corresponding new CHRGs signature was an independent prognostic indicator of lung adenocarcinoma and validated successfully in the external dataset GSE30219.

Copper is a vital trace element involved in key biochemical processes in the human body, but high levels can lead to health problems, including cancer-related processes^12^. The relationship between copper and cancer can be analyzed from two perspectives: cuproptoisis and copper homeostasis^13^. Studies indicate that elevated copper levels can stimulate the growth and invasion of cancer cells while impairing immune system function. Copper can also influence cancer pathophysiology by affecting mechanisms like DNA repair enzymes and apoptosis regulation^14^. On the other hand, cuproptoisis is a specific form of cell death triggered by copper ions and has potential applications in cancer treatment. It encompasses both apoptosis and autophagy induced by copper ions^15^. Research suggests that certain cancer cells exhibit greater sensitivity to copper ions, while normal cells show better tolerance. This knowledge offers the possibility of selectively targeting and eliminating cancer cells by inducing cuproptoisis or utilizing copper ion-related compounds, while minimizing harm to normal cells^16^. In our study, we identified several new potential influencing factors related to cuproptoisis and copper homeostasis which were differential expressed between normal lung cell line and lung adenocarcinoma cell lines. *MELTF* (Melanotransferrin) is a protein-coding gene involved in iron cellular uptake, which has similarity of sequence and iron-binding properties with members of the transferrin superfamily^17^. The function of protein encoded by *SEC14L3* (*SEC14* Like Lipid Binding 3) is uncertain, Gene Ontology annotation for *SEC14L3* includes transporter activity and lipid binding function, which also occurs in the process of cuproptosis^18^. CNTNAP2 belongs to a group of genes associated with human diseases and primarily participates in cell proliferation, adhesion, and various neurodevelopmental disorders. However, no literature has investigated its possible role in lung cancer^19^. The protein encoded by RHCG is primarily associated with cell ion homeostasis and protein binding, and has been found to be linked to several types of cancers. However, its specific role in either promoting or inhibiting cancer remains unclear, and there is limited research on its involvement in lung cancer^20^. Presently, there is still a significant scope for exploring the functions and mechanisms of these CHRGs, which requires further rigorous validation through experimental research.

Our study revealed the distinct biological and clinical significance of the CHRGs signature. Patients belonging to the CHRGs cluster-A exhibited a higher concentration within the high-risk group, indicating a poorer prognosis. Additionally, both the CHRGs cluster-A and the high-risk group were enriched in pathways associated with the malignant progression of the tumor. Furthermore, it was observed that patients in the high-risk group made significant contributions to the cuproptosis-associated pathway, as well as growth and metabolic pathways such as cell cycle, aging, and glycolysis pathways. This indicates heightened energy supply activity and more robust metabolic and apoptotic processes. Patients in the low-risk group exhibited a more active tumor microenvironment and a higher immune infiltration score, which may lead to a greater benefit from immunotherapy^21^. In addition, the risk scores showed a significant correlation with the function of antigen presenting cell (APC) co-inhibition, MHC I, and dendritic cell content. The risk scores displayed a positive correlation with the levels of anti-tumor cells like NK cells and macrophages. Conversely, they exhibited a negative correlation with immune cell contents, including B cells and neutrophils. This suggests that the high-risk group may exhibit a tumor microenvironment (TME) that is characterized by an active anti-tumor effect along with some degree of immunosuppression^22,23^. These results explained why the low-risk patients were mainly distributed in C3 (inflammatory) immune-type with better prognosis, while high-risk patients were predominantly found in the more aggressive immune-type C1 (wound-healing) and C2 (IFN-γ-dependent) subtypes^24^.

Indeed, the relationship between cuproptosis, copper homeostasis, and immunotherapy is complex and multifaceted. The effectiveness of immunotherapy can be highly variable due to the influence of factors such as copper ion concentration levels, specific immune cell types, and tumor characteristics. Interestingly, our study showed that both TIDE and IPS immune scores indicated a more favorable response to immune checkpoint inhibitor treatment in the low-risk group. While the precise effect of cuproptosis on immune efficacy has not been experimentally demonstrated, this underlines the significance of conducting further research to ascertain these relationships. The ultimate goal is to enhance our understanding of these connections, in order to optimize the outcomes of immunotherapy and develop more efficient treatment strategies. In addition to that, we employed a combination of two algorithms, Oncopredict and pRRophetic, to examine potential variations in the efficacy of different risk groups when utilizing conventional chemotherapy and novel targeted drugs^25^. At the single-cell level, our findings revealed that the CHRGs signature was predominantly concentrated in cancer epithelial cells and macrophages. Furthermore, cell populations exhibiting elevated distribution levels of CHRGs were enriched in P450-related drug metabolism pathways and displayed a silenced tumor microenvironment. This finding further strengthens the close association between CHRGs signature, chemical drug metabolism, and the potential benefits of immunotherapy. The cells exhibiting high expression of the CHRGs signature were also found to be enriched in functions related to metal ion homeostasis and lipid transport. These findings align with the characteristics of cuproptosis and copper homeostasis, which are consistent with our transcriptome analysis results at the tissue level.

There are several limitations to the study. First, the data we analyzed came from public data sets, the conclusions still need to be verified in multi-centers before being applied to clinical practice and strategy. Secondly, the differences we discussed were mainly among different samples, and it was difficult to consider the heterogeneity within tumors. In addition, given the possible deviation arising from different standardized processes, our model and conclusions are applicable to data generated based on next-generation sequencing (NGS) techniques with TPM standardized process. Finally, the specific biological mechanisms of cuproptosis and copper homeostasis in LUAD need to be further investigated in vivo and in vitro.

## Conclusion

In summary, our study identified new genotyping clusters related to cuproptosis and copper homeostasis, a prognostic signature based on 10 CHRGs (*LINC00858*, *INHA*, *LCAL1*, *SEC14L3*, *CNTNAP2*, *MELTF*, *RHCG*, *TM4SF4*, *NTSR1*, *PTX3*), and a novel clinical nomogram that can predict the prognosis of LUAD patients. This study also contributes to enhance the understanding of the potential impact of cuproptosis and copper homeostasis related genes in regulating cell pathways, influencing immune cell infiltration level, tumor mutation patterns, and antitumor drug selection.

## Materials and methods

### Data collection and pretreatment

Counts and transcripts per kilobase per million mapped reads (TPM) format transcriptomic data of 539 LUAD samples and 59 normal samples with corresponding clinical information were acquired from The Cancer Genome Atlas (TCGA) (https://portal.gdc.cancer.gov/, accessed on 13 October 2022). After removing normal samples and the samples with survival time equal 0 or deficient of survival information, 503 tumor samples were obtained. Lung cancer microarray data set GSE30219 based on platform GPL570 (Affymetrix Human Genome U133 Plus 2.0 Array), derived from the Gene Expression Omnibus (GEO) database (https://www.ncbi.nlm.nih.gov/geo/, accessed on 20 October 2022), 280 tumor samples containing complete clinical information (overall time, survival status, T, M, N, age, gender) were selected for the validation of the prognostic model. To calculate the single-cell distribution level of CHRGs, we selected a GPL18573-based (Illumina NextSeq 500, Homo sapiens) scRNA-seq dataset GSE183219 with 15 lung cancer samples and a GPL20795-based (HiSeq X Ten, Homo sapiens) scRNA-seq dataset GSE203360 with 3 pairs of lung cancer and para-cancer samples. The somatic mutation data of the LUAD cohort were obtained from TCGA, and the data of copy number variations were obtained from the UCSC Xena database (http://xena.ucsc.edu/, accessed on 16 November 2022)^26^. Tumor immune dysfunction and exclusion (TIDE) score of LUAD were acquired from TIDE website (http://tide.dfci.harvard.edu/, accessed on 25 October 2022), immunophenoscore (IPS) was acquired from The Cancer Immunome Atlas (TCIA) database (https://tcia.at/home, accessed on 25 October 2022)^27,28^. 19 genes related to cuproptosis were derived from the previous literatures. We collected 14 pathways related to copper homeostasis in Molecular Signatures Database (MSigDB) of GSEA website (https://www.gsea-msigdb.org/gsea/msigdb/index.jsp, accessed on 19 September 2022), and obtained 150 copper homeostasis related genes from them^29^.

### Identification of cuproptosis subtypes and copper homeostasis subgroups

We used R-package ‘ConsensusClusterPlus’ to identify the subtypes based on 19 cuproptosis related genes and k-means consensus clustering algorithm, repeated 1000 times to ensure clustering stability^30^. The copper homeostasis score of each sample was calculated using ssGSEA with R-package ‘GSVA’ based on the 150 genes derived from 14 copper homeostasis pathways, and the samples were grouped to high- and low-subgroup according to the median score^31^. Survival analysis between different subtypes and subgroups were based on the Kaplan–Meier analysis and two-sided log-rank test with R package ‘Survminer’. Differential analysis was conducted between cuproptosis subtypes and between copper subgroups based on counts of genes using R-package ‘Deseq2’, genes with |Log2 fold change|> 1 and p-adjusted < 0.05 were considered to be significantly different.

### Weighted gene co-expression network analysis and hub gene identification

To further explore the key genes associated with cuproptosis or copper homeostasis, R-package ‘WGCNA’ was performed on total 36,863 mRNAs and lncRNAs in 503 LUAD samples^32^. In this process, the suitable power exponent is selected to convert the adjacency matrix (AM) into the topological overlap matrix. Then, correlation analysis was conducted between the gene consensus modules and cuproptosis subtypes as well as copper homeostasis score. Modules with strong correlation and p-value < 0.05 were selected, the function ‘chooseTopHubInEachModule’ was used to identify hub genes in the selected modules for subsequent analysis.

### Identification of CHRGs related clusters

Cuproptosis and copper homeostasis related genes, differential genes between cuproptosis subtypes and between copper homeostasis subgroups, WGCNA hub genes were combined and selected as candidate CHRGs. Subsequently, differential analysis was conducted between 539 LUAD samples and 59 normal samples based on gene counts using R package ‘Deseq2’, genes with p-adjusted < 0.01 and |Log2 fold change|> 2 were identified as differentially expressed genes (DEGs). In addition, the gene symbols in GPL570 which annotated microarray chip data set GSE30219 was obtained. The intersection of candidate CHRGs, DEGs and annotated gene symbols was identified as CHRGs. K-means consensus clustering algorithm was performed on 503 LUAD samples based on CHRGs to identify CHRGs clusters.

### Construction and validation of CHRGs related prognostic model

In order to determine the independent prognostic CHRGs, we conducted a multivariate cox regression analysis for CHRGs. These independent prognostic genes were further included in the least absolute shrinkage and selection operator (LASSO) regression to reduce model parameters and collinearity based on the R package ‘glmnet’. Ten-fold cross validation method was used to determine the penalty coefficient ‘λ’ of regression model. Then, based on R package ‘survminer’ and Akaike information criterion (AIC), step-to-step regression method was used to determine the Cox proportional hazard model parameters when AIC value was the minimum^33^. The risk score of each sample was calculated according to the gene expression and regression coefficient, all samples were grouped according to the median score. The predictive ability of risk groups and clinical factors under 1, 3 and 5 years of survival was calculated using R package ‘timeROC’ and the independent prognostic factors of OS were identified by multivariate regression analysis. Finally, a nomogram includes independent prognostic factors was constructed based on R package ‘RMS’. The microarray chip dataset GSE30219 was used for external validation.

### Functions and pathways enrichment analysis

For the differential genes of cuproptosis subtypes and copper homeostasis subgroups, the functional enrichment analysis was based on Gene Ontology(GO) enrichment analysis by R-package ‘clusterProfiler’, and the pathway enrichment analysis was based on the online database Metascape (www.metascape.org/, accessed on 1 November 2022)^34,35^. The enrichment analysis based on R-package ‘GSVA’ was used to identify the enriched pathways of different CHRGs clusters. Gene set enrichment analysis (GSEA) software (http://www.gsea-msigdb.org/gsea/downloads.jsp, Version 4.3.2, Jill P. Mesirov and Pablo Tamayo, Principal Investigator, UC San Diego and Broad Institute) was used to identify pathways and functions enriched in different risk groups, the background gene set for functional analysis was ‘c5.go.v2022.1.Hs.symbols.gmt’, and the pathway gene set was ‘c2.all.v2022.1.Hs.symbols.gmt’, 1000 random sample permutations for stability^36^. The p-value < 0.05 was considered statistically significant.

### Comprehensive analysis of biological significance of CHRGs signature

The function ‘Cell-type identification by estimating relative subsets of RNA transcripts (CIBERSORT)’ was used to calculate the relative contents of various cell types in the samples^37^. The ssGSEA based on R-package ‘GSVA’ was used to estimate the level of immune cell infiltration and the score of immune function activity of the samples^38,39^. The R-package ‘Estimate’ calculated the tumor microenvironmental immune cell score and stromal cell score of each sample. The R package ‘Maftools’ was used to analyze the available MAF-formatted somatic mutation data of the LUAD cohort acquired from TCGA^40^. The immunophenotyping data of pan-cancer samples were obtained from the NCI Genomic Data Commons official website (GDC, https://portal.gdc.cancer.gov, accessed on 3 November 2022), which is used to identify the immune types of LUAD samples. Spearman correlation analysis was used to calculate the correlation between risk score and the expression levels of genes related to immune checkpoint, cell cycle and apoptosis.

### CHRGs signature predicts immunotherapy efficacy and anti-tumor drug sensitivity

We obtained TIDE score of LUAD cohort in TCGA from TIDE database, combined with IPS acquired from TCIA database to jointly evaluate the differences in the efficacy of immunotherapy in different risk groups. The R package ‘Oncopredict’ was used to calculate the drug sensitivity of each sample to different antitumor drugs for screening suitable antitumor drugs and novel small-molecule drugs associated with lung cancer^41^. In addition, we acquired drug targeting pathways from the genomics of drug sensitivity in cancer (GDSC) (https://www.cancerrxgene.org/, accessed on 15 November 2022), Spearman correlation analysis was then used to calculate the correlation coefficient between risk score and drug IC50 value calculated based on R-package ‘pRRophetic’^42^.

### Single cell RNA sequencing analysis reveals the distribution of CHRGs signature on cell levels

The processing of scRNA-seq data of GSE183219 and GSE203360 was based on R package ‘Seurat’^43^. In the quality control process, the cells with the gene features more than 500, the count -value more than 1000 and less than 20,000, and the percentage of mitochondrial genes less than 5% were reserved. The function ‘NormalizeData’ was used to normalize the data, and the function ‘VariableFeatures’ was used to find the highly variable genes. Principal component analysis (PCA) and uniform manifold approximation and projection analysis (UMAP) were used for dimensionality reduction and clustering of the homogenized data. The 'FindAllMarkers' function was used to identify differential markers between different UMAP clusters with the standard of | Log2 fold change |< 0.25 with p-adjusted < 0.05 and ‘min.pct’ was equal to 0.3. According to differentially expressed genes, cell clusters were annotated based on R-package’SingleR’ and the gene markers reported as previous in CellMaker database (http://xteam.xbio.top/CellMarker/, accessed on 21 November 2022). The area under curve (AUC) of CHRGs signature in each cell was calculated by the R package ‘AUCell’, and the cells above the AUC threshold were identified as CHRGs signature enriched cells^44^. GSEA was conducted to reveal the enriched pathways and functions of the CHRGs signature enriched cells.

### Cell culture and real time quantitative PCR (RT-qPCR)

Human lung adenocarcinoma cell line PC-9 (Cat# CL-0668), H1975 (Cat# CL-0298) and human bronchial epithelioid cell line 16-HBE (Cat# CL-0249) were purchased from Procell. Under rigorous sterile conditions at 37 °C and 5% CO2, cells were incubated in RPMI-1640 medium (Cellmax, Cat# CGM112.05) containing 10% fetal bovine serum (Procell, Cat# 164210-50) and 1% penicillin/streptomycin combination (Beyotime, Cat# C0222). RNAiso Plus (Takara, Cat# 9108) was used to extract total RNA from cells. With the use of the Hifair III 1st Strand cDNA Synthesis Super Mix kit (YEASEN, Cat# 11141ES), RNA was reverse transcribed into cDNA. β-actin was used as a reference, RT-qPCR was utilized to detect the expression of mRNAs and lncRNAs linked to cuproptosis and copper hemostasis using Hieff qPCR SYBR Green Master Mix (YEASEN, Cat# 11203ES). Table 1 lists the primers for these mRNAs and lncRNAs. Each sample was examined three times and the 2−(△CT sample−△CT control) was employed to determine the transcriptional levels of mRNA or lncRNA.

**Table 1:** Primer set of CHRGs for RT-qPCR.

Name	Forward primer (5′–3′)	Reverse primer (5′–3′)
CNTNAP2	CTTTGGCAATCGGAAGCAGAT	GAGCATCCGGTATTGGGTCAC
INHA	TTTTCCCAGCCACAGATGCC	CAGGGGCTCAGAGCTATTGG
LINC00858	GTCCCCACCTGGATCTTGTG	GGGGTGTGACAAGTGCTCAT
MELTF	GAGCCCCCTGGAGAGATACT	GAAGCGTCTTCCCATCCGTG
NTSR1	TCCTGAACACCATCATCGCC	GACCACTGCACGTAGGACG
PTX3	GCTCTCTGGTCTGCAGTGTT	CTTGTCCCATTCCGAGTGCT
TM4SF4	GGTCTACTGGGAGATGCCTG	CCTGCCCTGGGAGCCTA
SEC14L3	CCTGCCCAACCCTGATGATT	TCTCCCGTGTCCCTTTCTCT
RHCG	AGGAAGCGGTGTCTTGATGAT	ATCGTGGAGGTGAACATCGC

### Statistical analysis

In the part of bioinformatic analysis, R software (https://cran.r-project.org/src/base/R-4/, Version 4.0.5, Ross Ihaka and Robert Gentleman, Auckland, New Zealand) was used for data analysis and picture drawing process. For survival analysis, Kaplan–Meier analysis and two-sided log-rank test were used for inter-group comparison. Welch's t test was used to compare the mean value between two groups of samples, and median values were compared by Wilcoxon rank-sum test. Spearman analysis was used for correlation analysis. The GraphPad Prism 8 software (https://www.graphpad.com/, Version 8.0.1, San Diego, California USA) was used to analyze the RT-qPCR data and t-test was used to determine the p-value, the findings were presented as Mean ± SEM. The symbols ‘ns’, ‘*’, ‘**’, ‘***’, ‘****’ correspond to ‘p-value ≧ 0.05’, ‘p-value < 0.05’, ‘p-value < 0.01’, ‘p-value < 0.001’, and ‘p-value < 0.0001’, respectively. A two-sided p-value < 0.05 was considered statistically significant.

### Supplementary Information

Supplementary Information.

## Data Availability

The analysis data in the paper can be found in TCGA database (https://portal.gdc.cancer.gov/), GEO database (https://www.ncbi.nlm.nih.gov/geo/), UCSC Xena database (http://xena.ucsc.edu/), and MSigDB (https://www.gsea-msigdb.org/gsea/msigdb/index.jsp). The secondary research data were obtained from Metascape (www.metascape.org/), TIDE database (http://tide.dfci.harvard.edu/), TCIA database (https://tcia.at/home), GDSC database (https://www.cancerrxgene.org/), and PubChem database (https://pubchem.ncbi.nlm.nih.gov/). All the obtained data in this study is publicly available and complied with the statements and norms of the official websites, there were no ethical problems or copyright conflicts.
