# Supplementary material for: Comprehensive analysis of cuproptosis and copper homeostasis genotyping and related immune land scape in lung adenocarcinoma
Source: Sci Rep. 2023 Oct 2;13:16554. doi: 10.1038/s41598-023-43795-3 (PMC10545825; doi:10.1038/s41598-023-43795-3)
Supplement: Supplementary file 1 — Supplementary Information. [file 41598_2023_43795_MOESM1_ESM.docx]

**SUPPLEMENTARY MATERIALS**

**Supplementary Figure 1**

**
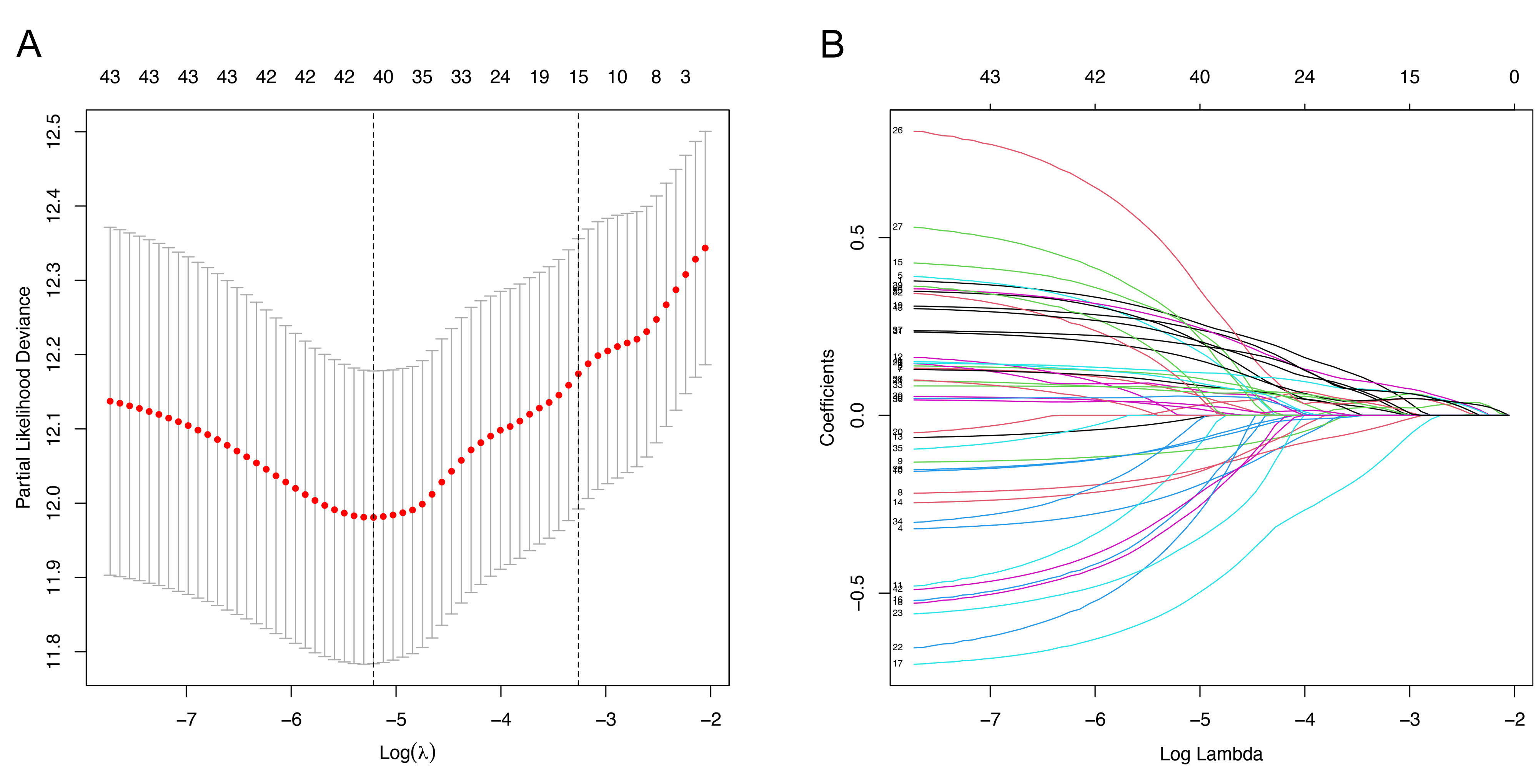
**

**Supplementary Figure 1.** Selection of candidate genes for the Cox proportional hazard model based on LASSO regression. (**A**)The correlation between the partial likelihood deviance and log(λ), lambda.min (left dashed line) is the value of λ that results in the smallest mean of the target parameter and lambda.1se (right dashed line) refers to the λ value within the variance range of lambda.min to obtain the simplest model. (**B**) Coefficient of the parameters were reduced to 0.

**Supplementary Figure 2**

**
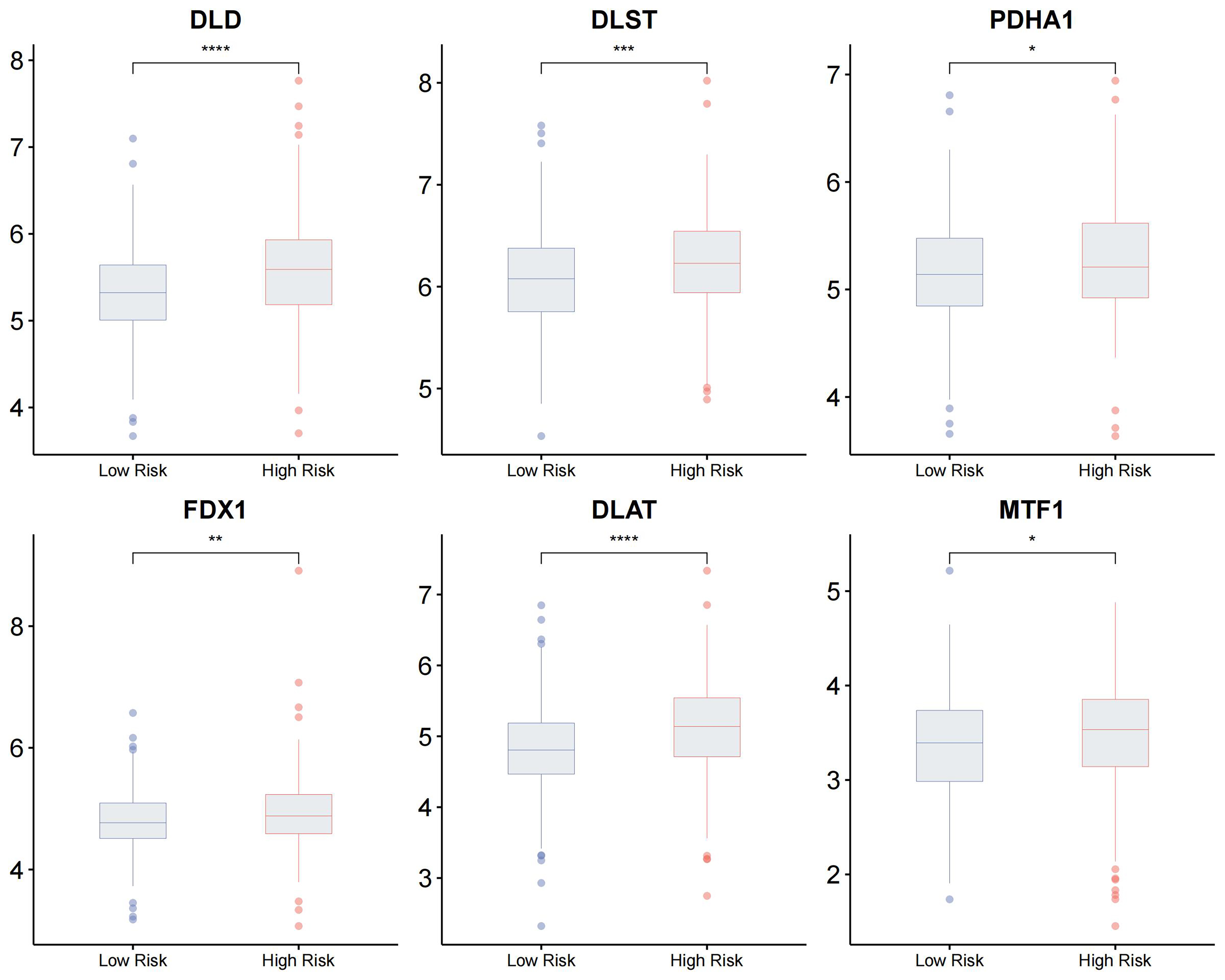
**

**Supplementary Figure 2.** Six cuproptosis related genes with significant differences in expression levels between different risk groups.

**Supplementary Table 1**

**Supplementary Table 1.** 10-gene signature of prognostic model.

| **Gene** | **Coef** | **HR** | **HR.95L** | **HR.95H** | **P value** |
| --- | --- | --- | --- | --- | --- |
| **LINC00858** | 0.213246 | 1.237689 | 1.055306 | 1.451593 | 0.008747 |
| **INHA** | 0.080131 | 1.083429 | 1.008996 | 1.163353 | 0.027344 |
| **LCAL1** | -0.11938 | 0.887468 | 0.819405 | 0.961184 | 0.003364 |
| **SEC14L3** | -0.50269 | 0.604899 | 0.422372 | 0.866306 | 0.006086 |
| **CNTNAP2** | 0.20836 | 1.231657 | 1.073619 | 1.412958 | 0.002941 |
| **MELTF** | 0.182404 | 1.200099 | 1.061608 | 1.356656 | 0.00355 |
| **RHCG** | 0.108525 | 1.114633 | 1.004236 | 1.237165 | 0.04141 |
| **TM4SF4** | 0.081644 | 1.08507 | 1.026217 | 1.147298 | 0.004111 |
| **NTSR1** | 0.111401 | 1.117843 | 0.994056 | 1.257045 | 0.062828 |
| **PTX3** | 0.185254 | 1.203524 | 1.046129 | 1.384601 | 0.009581 |

**Supplementary Table 2**

**Supplementary Table 2.** Clinical baseline characteristics in different cohorts and risk groups.

|  |  | | **TCGA** | |  | |  | | **GEO** | |  |
| --- | --- | --- | --- | --- | --- | --- | --- | --- | --- | --- | --- |
|  | Low Risk | | High Risk | | Total | | Low Risk | | High Risk | | Total |
|  | (N=158) | | (N=179) | | (N=337) | | (N=144) | | (N=136) | | (N=280) |
| **Gender** |  | |  | |  | |  | |  | |  |
| FEMALE | 87 (55.1%) | | 85 (47.5%) | | 172 (51.0%) | | 24 (16.7%) | | 17 (12.5%) | | 41 (14.6%) |
| MALE | 71 (44.9%) | | 94 (52.5%) | | 165 (49.0%) | | 120 (83.3%) | | 119 (87.5%) | | 239 (85.4%) |
|  |  | |  | |  | |  | |  | |  |
| **Age (years)** | |  | |  | |  | |  | |  | |
| Mean (SD) | 64.5 (10.3) | | 65.1 (10.3) | | 64.8 (10.3) | | 60.9 (10.9) | | 61.5 (12.1) | | 61.2 (11.4) |
| Median [Min, Max] | 65.5 [33.0, 86.0] | | 66.0 [40.0, 87.0] | | 66.0 [33.0, 87.0] | | 61.5 [25.0, 84.0] | | 63.0 [15.0, 82.0] | | 62.0 [15.0, 84.0] |
|  |  | |  | |  | |  | |  | |  |
| **Stage** |  | |  | |  | |  | |  | |  |
| Stage I | 101 (63.9%) | | 72 (40.2%) | | 173 (51.3%) | | / | | / | | / |
| Stage II | 37 (23.4%) | | 46 (25.7%) | | 83 (24.6%) | | / | | / | | / |
| Stage III | 16 (10.1%) | | 44 (24.6%) | | 60 (17.8%) | | / | | / | | / |
| Stage IV | 4 (2.5%) | | 17 (9.5%) | | 21 (6.2%) | | / | | / | | / |
|  |  | |  | |  | |  | |  | |  |
| **T** |  | |  | |  | |  | |  | |  |
| T1 | 62 (39.2%) | | 40 (22.3%) | | 102 (30.3%) | | 89 (61.8%) | | 74 (54.4%) | | 163 (58.2%) |
| T2 | 79 (50.0%) | | 111 (62.0%) | | 190 (56.4%) | | 34 (23.6%) | | 31 (22.8%) | | 65 (23.2%) |
| T3 | 11 (7.0%) | | 17 (9.5%) | | 28 (8.3%) | | 13 (9.0%) | | 18 (13.2%) | | 31 (11.1%) |
| T4 | 6 (3.8%) | | 11 (6.1%) | | 17 (5.0%) | | 8 (5.6%) | | 13 (9.6%) | | 21 (7.5%) |
|  |  | |  | |  | |  | |  | |  |
| **M** |  | |  | |  | |  | |  | |  |
| M0 | 154 (97.5%) | | 162 (90.5%) | | 316 (93.8%) | | 142 (98.6%) | | 133 (97.8%) | | 275 (98.2%) |
| M1 | 4 (2.5%) | | 17 (9.5%) | | 21 (6.2%) | | 2 (1.4%) | | 3 (2.2%) | | 5 (1.8%) |
|  |  | |  | |  | |  | |  | |  |
| **N** |  | |  | |  | |  | |  | |  |
| N0 | 119 (75.3%) | | 94 (52.5%) | | 213 (63.2%) | | 110 (76.4%) | | 83 (61.0%) | | 193 (68.9%) |
| N1 | 27 (17.1%) | | 45 (25.1%) | | 72 (21.4%) | | 22 (15.3%) | | 29 (21.3%) | | 51 (18.2%) |
| N2 | 12 (7.6%) | | 39 (21.8%) | | 51 (15.1%) | | 10 (6.9%) | | 17 (12.5%) | | 27 (9.6%) |
| N3 | 0 (0%) | | 1 (0.6%) | | 1 (0.3%) | | 2 (1.4%) | | 7 (5.1%) | | 9 (3.2%) |
|  |  | |  | |  | |  | |  | |  |
| **Survival State** |  | |  | |  | |  | |  | |  |
| Death | 44 (27.8%) | | 92 (51.4%) | | 136 (40.4%) | | 89 (61.8%) | | 100 (73.5%) | | 189 (67.5%) |
| Alive | 114 (72.2%) | | 87 (48.6%) | | 201 (59.6%) | | 55 (38.2%) | | 36 (26.5%) | | 91 (32.5%) |
